# Underlying Conditions in Community-associated Clostridioides difficile Infections in Davidson County, Tennessee

**DOI:** 10.1017/ash.2024.187

**Published:** 2024-09-16

**Authors:** Marissa Turner, Kristina McClanahan, Frederick Thompson, Zelda Foster, Raquel Villegas

**Affiliations:** Tennessee Department of Health; TN DEPT OF HEALTH CEDEP/HAI; state of TN

## Abstract

**Background:** Clostridioides difficile infections (CDI) are a crucial public health threat becoming a worldwide problem. In 2017, there were 223,900 incident cases and 12,800 deaths in the United States. Underlying conditions, such as diabetes mellitus (DM), put individuals at a greater risk for developing an infection. Whereas CDI was once believed to be mostly healthcare-associated, increasing evidence points to transmission in community settings (CA). We investigated characteristics of CA CDI and associations between pre-existing conditions and CA incident CDI cases using data from Tennessee’s CDI surveillance program, an active population- and laboratory-based surveillance system conducted through CDC’s Emerging Infections Program. CA incident CDI case data were downloaded from the Incident Case Management System from 2017 to 2021. Count and percentages were determined for each underlying condition, number of underlying conditions, and biological sex. Chi-square analyses determined associations between underlying conditions and sex. Statistical analyses were conducted using SAS v9.4. 2,326 CA incident CDI cases were identified from the catchment area. The case rates per 100,000 population between 2017 and 2021 were 79.7, 81.9, 73.7, 50.7, and 49.6. A total of 39% of the cases were 65 years or older. Most cases were women (64.8%). The overall prevalence for any underlying condition among CA CDI cases was 67.4%. A total of 29.4% of incident cases had one condition, 18.5% had two conditions, and 19.4% had three or more conditions. The most frequently reported pre-existing conditions was DM (22.9%) and gastrointestinal disease (21.7%). We looked at the prevalence of underlying conditions separated in men and women. Men with CA CDI were more likely to have chronic kidney disease (CKD) (19.1% vs 12.7%), DM (26.0% vs 21.2%), immunocompromised conditions (6.4% vs 3.6%), liver diseases (6.5% vs 2.8%), and plegias (1.0% vs 0.2%) than women with CA CDI. Women with CA CDI were more likely to have chronic lung diseases (17.4% vs 12.6%) and connective tissue diseases (4.9% vs 2.2%) than men with CA CDI. Although the incident CA CDI case rate in Davidson County decreased from 2018 to 2021, it remains a significant threat. In this analysis, underlying conditions in persons with CA CDI were highly prevalent. Men were more likely to have underlying conditions in general, and specifically CKD and DM, than women. Improving understanding of the prevalence of these conditions with CA CDI cases, along with their antibiotic use and community exposures, can help drive prevention strategies to mitigate CA CDI transmission.

